# Identification of galactosamine-(*N*-acetyl)-6-sulfatase (GALNS) as a novel therapeutic target in progression of nasopharyngeal carcinoma

**DOI:** 10.1007/s12672-023-00782-4

**Published:** 2023-09-14

**Authors:** Jin Zhang, Hong Ran, Zhen Wang, Peng Liu, Chenglin Kang, Xianhai Zeng, Shuqi Qiu, Peng Zhang

**Affiliations:** 1 Department of Graduate and Scientific Research, Zunyi Medical University Zhuhai Campus, Zhuhai, Guangdong China; 2 Department of Otorhinolaryngology, Longgang Otorhinolaryngology Hospital & Shenzhen Key Laboratory of Otorhinolaryngology, Shenzhen Institute of Otorhinolaryngology, 3004 Longgang Avenue, Shenzhen, NoGuangdong China; 3 Department of Otorhinolaryngology, The Second People’s Hospital of Yibin, Yibin, Sichuan China

**Keywords:** Nasopharyngeal carcinoma, GALNS, Progression, AKT, mTOR

## Abstract

Nasopharyngeal carcinoma (NPC) is a commonly diagnosed malignancy in southern China and southeast Asia. Previous studies have identified galactosamine-(*N*-acetyl)-6-sulfatase (GALNS) as a potential biomarker for multiple cancers. However, it is unknown whether GALNS plays a role in NPC development, and the underlying mechanisms remain unclear. In this study, we found that GALNS is overexpressed in NPC cell lines and tissues compared to the normal nasopharyngeal counterparts. Knocking down GALNS expression in the NPC cells significantly decreased their proliferation in vitro, and inhibited xenograft growth in a mouse model. Mechanistically, the anti-proliferative effect of GALNS silencing was the result of autophagy induction via the inhibition of PI3K–AKT–mTOR signaling pathway. Taken together, GALNS drives the progression of NPC via PI3K–AKT–mTOR signaling-mediated autophagy, and is therefore a promising therapeutic target.

**Supplementary Information:**

The online version contains supplementary material available at 10.1007/s12672-023-00782-4.

## Introduction

Nasopharyngeal carcinoma (NPC) is a common malignancy of the mucosal epithelium of the nasopharynx. The distribution of NPC differs significantly across ethnicities and geographical regions, and the highest incidence is seen in southern China and southeast Asia [1, 2]. Epstein Barr virus (EBV) infection, environmental influences, genetic predisposition and dietary habits are the key factors driving NPC progression [3]. Radiotherapy and chemotherapy are currently the standard treatment regimens for NPC, and can improve the 10-year survival rate of NPC patients in the early stage of the disease [4]. However, since early-stage NPC is asymptomatic, most patients are diagnosed in the advanced stage and respond poorly to therapies, resulting in shorter median survival. Therefore, it is critical to elucidate the signaling pathways underlying the progression of NPC in order to identify novel therapeutic targets and prognostic biomarkers.

The galactosamine-(*N*-acetyl)-6-sulfatase (GALNS) gene is located on chromosome 16q24.3, and was first purified from human placenta [5]. GALNS is a lysosomal enzyme that removes the 6-sulfate group from the non-reducing ends of chondroitin-6-sulfate and keratan sulfate [6]. GALNS mutation and deficiency lead to the development of the lysosomal storage disease Morquio A [7]. In contrast, Bhattacharya et al. detected significantly higher activity of GALNS in human prostate tumors compared to the normal tissues [8]. Wintergerst et al. reported that GALNS mRNA expression can predict the prognosis of head and neck squamous cell carcinoma patients after adjuvant radiotherapy or chemotherapy [9]. In addition, a recent study showed that GALNS is a reliable diagnostic biomarker for colon cancer, lung cancer, ovarian cancer and breast cancer [10]. Studies increasingly show that aberrant activity of lysosomal enzymes is a key driver of tumorigenesis [11–13]. However, the role of GALNS in NPC progression and the potential molecular mechanism are unclear.

Autophagy is a highly conserved self-catabolic process wherein misfolded or aberrant proteins and damaged organelles are degraded via the lysosomal pathway [14, 15]. It occurs in the cells of yeast, nematodes, drosophila and higher vertebrates [16, 17]. The process of autophagy is initiated with the formation of autophagosomes, which engulf the proteins and organelles, and then fuse with the lysosomes to form autolysosomes that degrade the cytoplasmic contents [18, 19]. Starvation, chemotherapeutic drugs, DNA damage and other cellular stresses can induce autophagy, which plays a critical role in cell survival or death [17, 20]. The general consensus is that autophagy suppresses tumor progression in the initial stages but supports cancer cell survival in the later stages [21, 22]. Zhu et al. reported that overexpression of MIR106A-5p accelerated the malignant transformation of NPC cells by inhibiting BTG3-mediated autophagy [23]. Furthermore, nitric oxide synthase 1 reduced autophagy and promoted the survival of NPC cells by *S*-nitrosylation of PTEN [24]. However, whether autophagy is involved in GALNS-mediated NPC development, or the involvement of other factors, remain unknown.

In this study, we found that GALNS is overexpressed in NPC tissues, and knocking down GALNS gene in NPC cells inhibited their proliferation in vitro and in vivo. Furthermore, GALNS downregulation induced autophagy via the AKT/mTOR signaling axis. This study is the first to establish an oncogenic role of GALNS in NPC, and its potential as a therapeutic target.

## Methods

### Cell culture

The human NPC cell lines CNE1, CNE2, HONE1, 5-8F, 6-10B and C666-1 were cultured in DMEM or RPMI 1640 medium supplemented with 10% fetal bovine serum (FBS), 100 U/ml penicillin and 100 μg/ml streptomycin. The immortalized nasopharyngeal epithelium cell line NP69 was cultured in serum-free keratinocyte growth medium supplemented with 5 μg/L epidermal growth factor and 50 mg/L bovine pituitary extract (Gibco). The cells were cultured at 37 °C in a humidified incubator with 95% O_2_ and 5% CO_2_.

### siRNA transfection

The cells were transfected with siRNAs using Lipofectamine™ RNAiMAX (Invitrogen) according to the manufacturer’s instructions. The siRNAs were synthesized by Shanghai GenePharma Co. Ltd and the sequences were as follows: siGALNS#1: 5′-CGCAAUGGCUUCUACACCATT-3′, siGALNS#2: 5′-GUCCGGGAGAUUGAUGACATT-3′. simTOR: 5′-GAGCAUGCCGUCAAUAAUAUUTT-3′. The cells were harvested 48 or 72 h after transfection, and total RNA or protein was isolated.

### Quantitative real-time PCR

Total RNA was isolated using the RNeasy Mini Kit (Qiagen) according to the manufacturer’s instructions. First-strand cDNA was synthesized using the RT Master Mix for qPCR kit (MCE). Quantitative real-time PCR was performed using SYBR Green qPCR Master Mix (MCE) on the Applied Biosystems 7500 FAST Real Time PCR system. The primer sequences were as follows: Actin forward 5′-CACCATTGGCAATGAGCGGTTC-3′, reverse 5′-AGGTCTTTGCGGATGTC CACGT-3′; GALNS forward 5′-AGCAGACCACGTTTGAAGGAGG-3′; reverse 5′-GTGGTGAAGAGGTCCATGATGC-3′;

### Cell viability assay

Cell viability was determined using the Cell Counting Kit-8 (CCK-8) reagent (MCE). Briefly, the NPC cells were seeded in 96-well plates and cultured for 0–72 h. After each time point, 10 μL CCK-8 reagent was added to each well and the cells were incubated for 2 h at 37 °C. The absorbance was measured at 450 nm using a multi-mode plate reader (Molecular Devices).

### Colony formation assay

The cells were seeded in a 6-well plate at the density of 500 cells/well and cultured for 2 weeks. After washing twice with PBS, the cells were fixed with 4% paraformaldehyde (PFA) for 15 min and stained with crystal violet. Colonies with more than 50 cells were counted.

### EdU assay

Edu assay was performed using the BeyoClick™ EdU Cell Proliferation Kit with Alexa Fluor 488 (Beyotime Biotech) according to the manufacturer’s protocol. Briefly, the cells were incubated with EdU for 2 h, fixed with 4% PFA for 15 min, and permeabilized with 0.3% Triton X-100 for 15 min. After incubating with the Click Reaction Mixture for 30 min in the dark, the cells were incubated with Hoechst 33342 for 10 min. Images were captured on a Leica TCS SP5 confocal microscope.

### Western blotting

Total protein was extracted from the cultured cells using RIPA lysis buffer (Beyotime Biotech), separated by 10% SDS-PAGE, and then transferred onto PVDF membrane (Millipore). After blocking with 5% BSA in TBST for 1 h at room temperature, the membranes were incubated overnight with primary antibodies at 4 °C, followed by HRP-conjugated secondary antibodies (Cell Signaling) at room temperature for 2 h. The protein bands were detected using the Pierce™ ECL Western Blotting Substrate (Thermo Scientific).

### Immunofluorescence staining

Cultured cells were fixed in 4% PFA (Sigma-Aldrich) for 15 min, and then blocked in 5% goat serum (Beyotime) with 0.1% Triton X-100 (Beyotime) in PBS for 30 min at room temperature. The cells were then incubated overnight with anti-GALNS antibody (1:200; Proteintech) at 4 °C, followed by Alexa Fluor-488 donkey anti-rabbit IgG (1:500) (Invitrogen) for 1 h at room temperature. After counterstaining with DAPI (Beyotime), the cells were viewed by confocal microscopy.

### Immunohistochemistry (IHC)

Tissue slides were deparaffinized, covered with 3% hydrogen peroxide for 10 min to block endogenous peroxidase activity, and then immersed in Improved Citrate Antigen Retrieval Solution (Beyotime) at sub-boiling temperature for 10 min. After blocking in 10% goat serum (Beyotime) with 0.2% Triton X-100 (Beyotime) in PBS for 1 h at room temperature, the slides were incubated with anti-GALNS antibody (1:200; Proteintech) at 4 °C in a humidified chamber. Subsequently, the sections were incubated with the GTVision III Detection System/Mo&Rb Kit (Gene Tech). Each specimen was scored according to the intensity of staining (0, none; 1, weak; 2, moderate; 3, strong) and the proportion of stained cells (0, 0%; 1, 1–24%; 2, 25–49%; 3, 50–74%; 4, 75–100%) according to the German semi-quantitative scoring system. The final immunoreactivity score was determined by multiplying the intensity with the positivity rate, and ranged from 0 to 12.

### Xenograft tumor model

All experiments (maximal tumor size/burden were permitted and not exceeded) were approved by the Animal Experimental Ethics Committee of Shenzhen Institute of Otorhinolaryngology and Use of Laboratory Animals published by the US National Institute of Health. The tumor model was established by subcutaneously injecting nude mice with CNE2 or HONE1 cells expressing the GALNS shRNA or negative control. The volume of the implanted tumor was measured using the formula:

$$ {\text{Volume}}\left( {{\text{mm}}^{{3}} } \right) = {\text{width}}^{{2}} \, \times \,{\text{length}}/{2}. $$

### Statistical analysis

Statistical analysis was performed using two-tailed Student’s t-test between two groups. Differences among three or more groups were compared by one-way ANOVA. Cell viability and tumor volume were analyzed by two-way ANOVA. All analyses were performed using the GraphPad Prism software version 5.0. Data were expressed as mean ± standard error of the mean (SEM) of at least three independent experiments. *P* value < 0.05 was considered statistically significant.

## Results

### GALNS is overexpressed in NPC tissues and cell lines

**Fig. 1:**
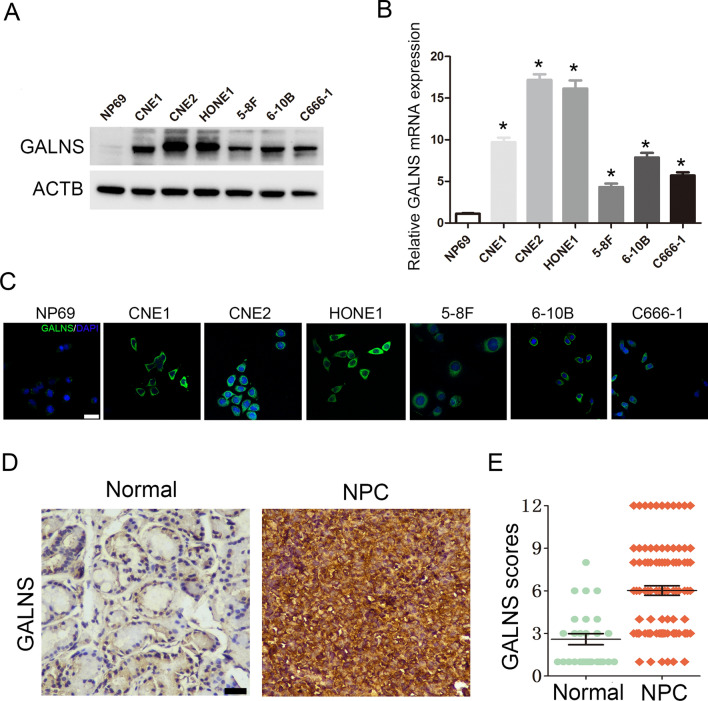
GALNS expression in NPC tissues and cell lines. **A** Immunoblot showing GALNS expression in NP69 cells and NPC cell lines. **B** Relative GALNS mRNA expression in NP69 cells and NPC cell lines. **C** Representative immunofluorescence images showing GALNS expression in NP69 cells and NPC cell lines Scare bar = 20 μm. **D**, **E** Representative images and scores of GALNS protein expression in normal nasopharyngeal (n = 27) and NPC tissues (n = 96). Scare bar = 50 μm. The data is the mean ± SEM of at least three independent experiments. *p < 0.05, versus NP69

To assess the possible involvement of GALNS in NPC, we compared its expression levels between a nasopharyngeal epithelial cell line (NP69) and multiple NPC cell lines (CNE1, CNE2, HONE1, 5-8F, 6-10B and C666-1). GALNS mRNA and protein expression were significantly higher in all NPC cell lines compared to that in the NP69 cells (Fig. 1A, B). A similar increase in GALNS protein expression was observed by immunofluorescence staining (Fig. 1C). To explore the clinical significance of GALNS expression, we further analyzed NPC tissues (n = 96) and nasopharyngeal tissues (n = 27) by IHC (Fig. 1D). As shown in Fig. 1E, the IHC scores were significantly higher in the NPC tissues relative to the normal nasopharyngeal tissues. Moreover, GALNS level was overexpressed in the HNSC tissues compared to that in the normal tissues through analysis of the data from The Cancer Genome Atlas (TCGA) (Additional file 1: Figure S1).Taken together, these results indicate that GALNS is overexpressed in NPC tissues and cell lines.

### GALNS knockdown suppressed the proliferation of NPC cells in vitro

**Fig. 2:**
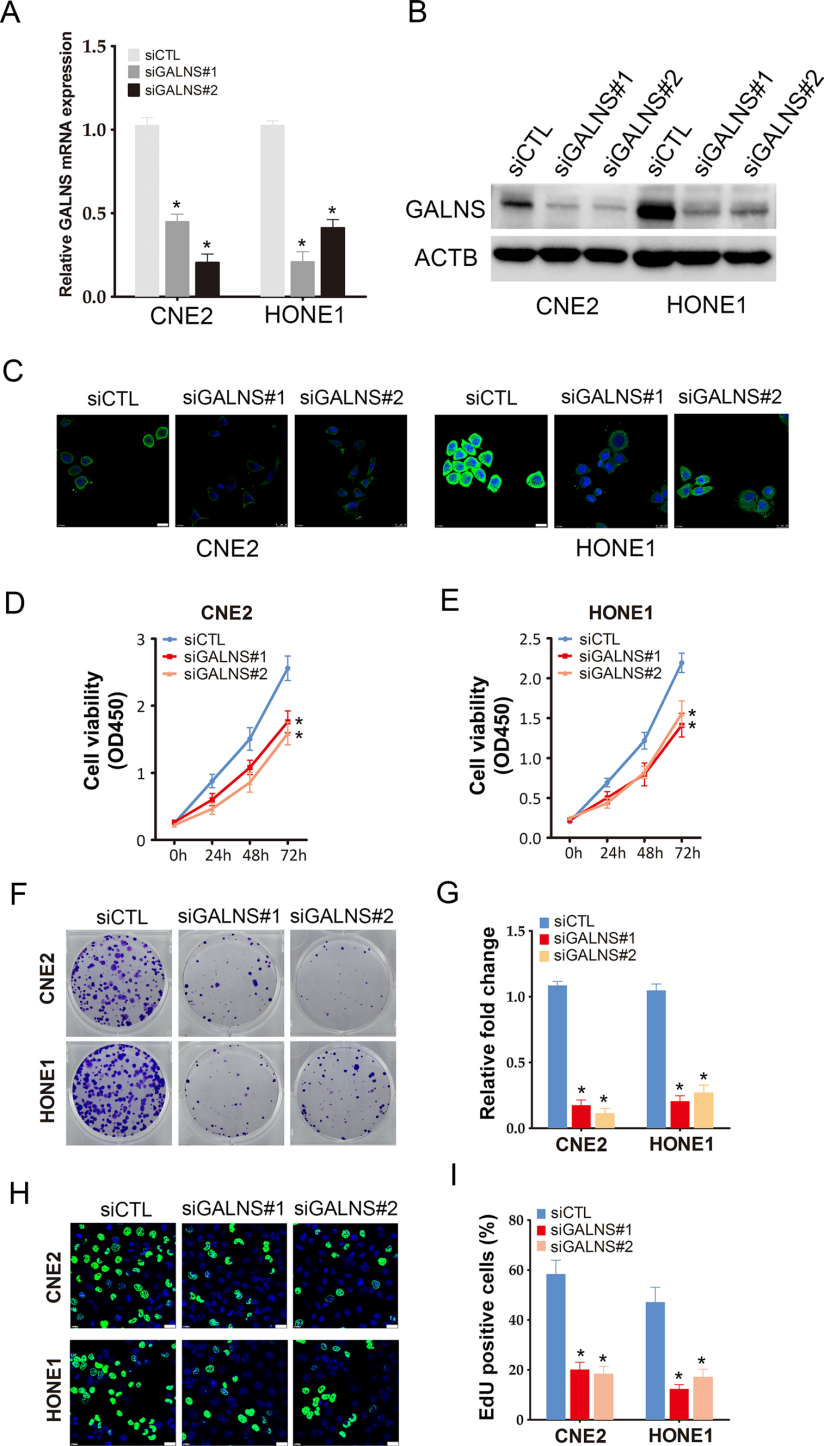
GALNS promotes NPC cells growth in vitro. **A**–**C** GALNS protein and mRNA expression in CNE2 and HONE1 cells transfected with siCTL, siGALNS#1 or siGALNS#2. **D**, **E** Viability of CNE2 and HONE1 cells transfected with siCTL, siGALNS#1 or siGALNS#2. **F**, **G** Representative images and number of colonies formed by CNE2 and HONE1 cells transfected with siCTL, siGALNS#1 or siGALNS#2. **H**, **I** Representative images and number of Edu-positive proliferative CNE2 and HONE1 cells transfected with siCTL, siGALNS#1 or siGALNS#2. The data represent the mean ± SEM of at least three independent experiments. Scare bar = 20 μm *p < 0.05, versus vehicle or siCTL

**Fig. 3:**
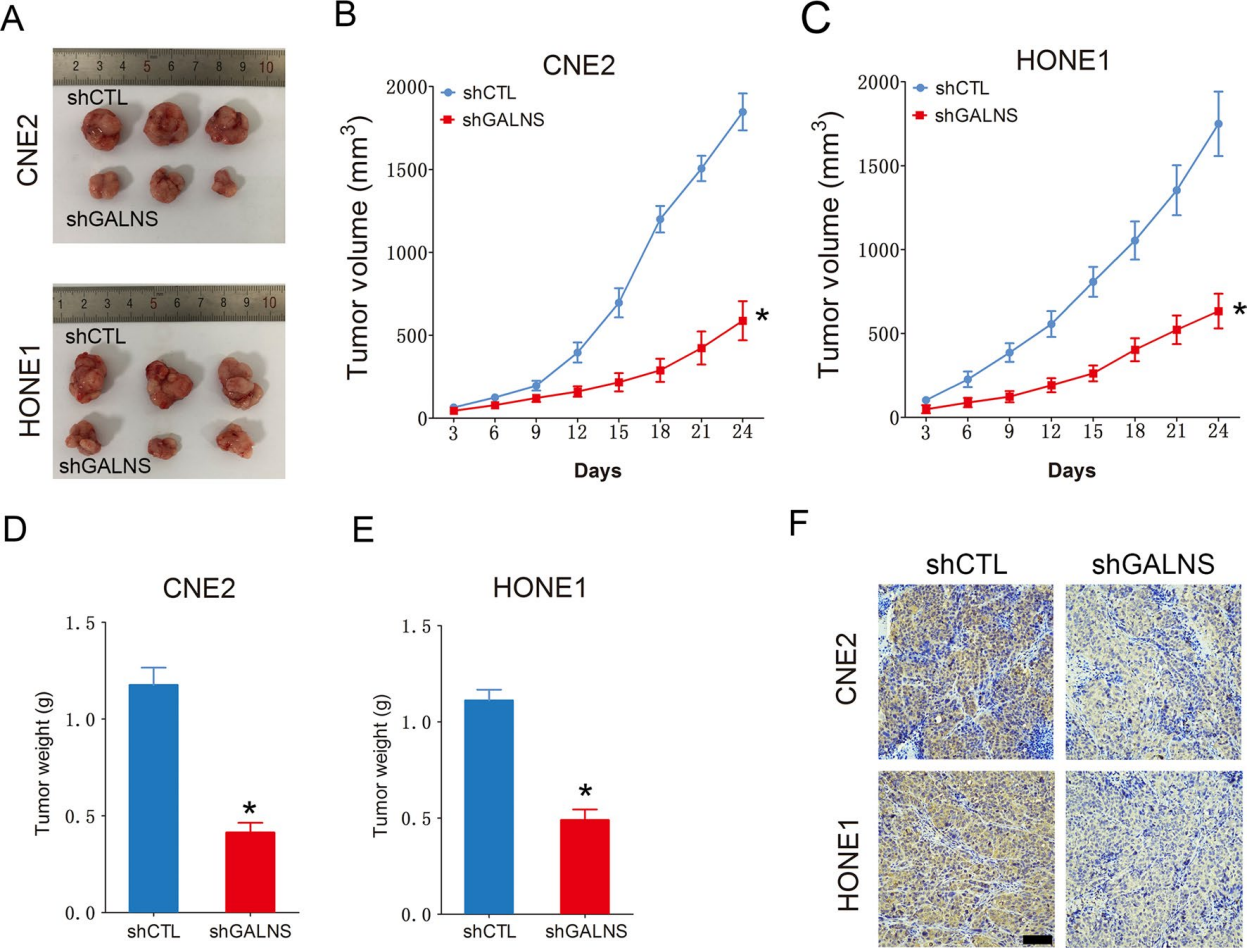
GALNS promotes NPC cells growth in vivo. **A** Representative images of the xenografts of NPC cells transfected with shCTL or shGALNS. **B**, **C** Average tumor volume in the indicated groups. **D**, **E** The tumor weight in the indicated groups. **F** Representative images of tumor tissues showing GALNS expression. Scare bar = 50 μm. The data represent the mean ± SEM of at least three independent experiments. *p < 0.05, versus shCTL

To further confirm the biological function of GALNS in NPC cells, we silenced the gene expression using two siRNAs and confirmed reduced expression of GALNS mRNA (Fig. 2A) and protein (Fig. 2B, C) in the CNE2 and HONE1 cells transfected with GALNS siRNAs compared to the controls. GALNS knockdown decreased the viability of CNE2 and HONE1 cells (Fig. 2D, E) and C666-1 cells (Additional file 1: Figure S2A) as well as their colony formation ability (Fig. 2F and Additional file 1: Figure S2B). As shown in Fig. 2G, cells with GALNS knockdown formed markedly fewer colonies compared to their respective controls. We also evaluated the effect of GALNS on cell proliferation by the EdU assay, wherein EdU-positive cells (green) are indicative of DNA synthesis (Fig. 2H). The number of proliferating EdU-positive cells decreased significantly in the GALNS-knockdown group compared to the control group (Fig. 2F). To assess the effect of GALNS silencing on NPC growth in vivo, a xenograft model was established by subcutaneously injecting CNE2 or HONE1 cells transfected with GALNS shRNA or control shRNA into nude mice. The GALNS-knockdown cells formed significantly smaller tumor size compared to control cells, as measured in terms of both volume (Fig. 3A–C) and weight (Fig. 3D, E). The low expression of GALNS protein was confirmed in the tumor tissues originating from the cells transfected with GALNS shRNA compared to that of the control group by immunohistochemistry (Fig. 3F).

### GALNS knockdown inhibited NPC growth via induction of autophagy

**Fig. 4:**
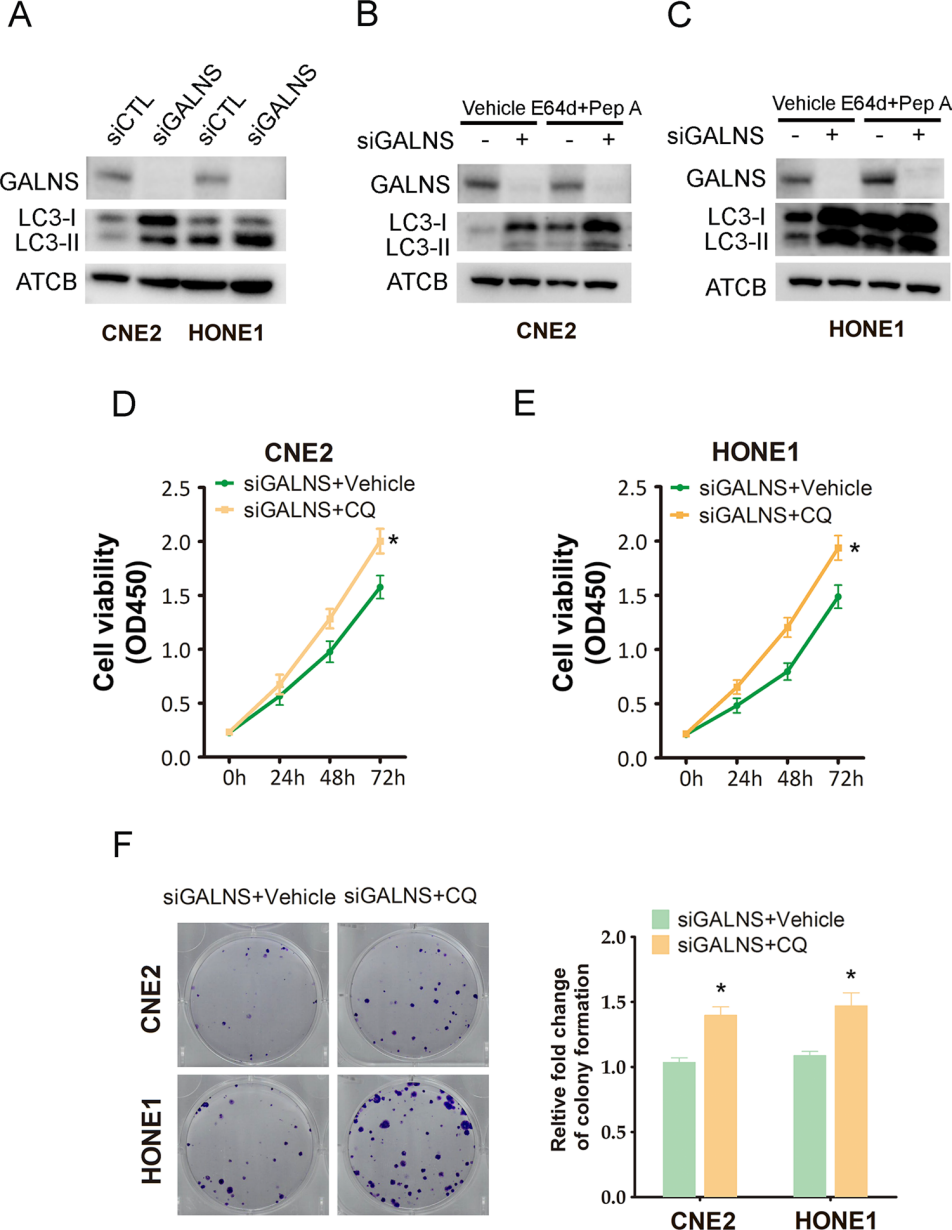
GALNS knockdown in NPC cells induced autophagy. **A** Immunoblot showing LC3-II protein expression in GALNS-knockdown CNE2 and HONE1 cells. **B**, **C** Immunoblot showing LC3-II expression in GALNS-knockdown CNE2 and HONE1 cells treated with the lysosomal protease inhibitors E64d + pepstatin A (Pep A). **D**, **E** Viability of GALNS-knockdown CNE2 and HONE1 cells following CQ treatment. **F** Representative images and number of colonies formed by the CQ-treated GALNS-knockdown CNE2 and HONE1 cells. The data represent the mean ± SEM of at least three independent experiments. *p < 0.05, versus siGALNS + Vehicle

The possible effect of GALNS downregulation on autophagy was evaluated by analyzing the expression of microtubule-associated protein 1 light chain 3 (LC3-I and LC3-II). LC3-II is an essential marker of autophagy that controls the maturation of autophagosomes. As shown in Fig. 4A, knocking down GALNS increased LC3-II expression. Moreover, the protease inhibitor E64d + Pep A augmented LC3-II levels in the GALNS-knockdown NPC cells, suggesting that silencing of GALNS can trigger a complete autophagic flux (Fig. 4B, C). Interestingly, the autophagy suppressor chloroquine (CQ) significantly enhanced the viability (Fig. 4D,E) and colony formation ability (Fig. 4F) of the GALNS-knockdown CNE2 and HONE1 cells. Taken together, our results suggest that the anti-proliferative effects of GALNS silencing on NPC cells are mediated via autophagy induction.

### GALNS knockdown induced autophagy in NPC cells via PI3K–AKT–mTOR signaling pathway

**Fig. 5:**
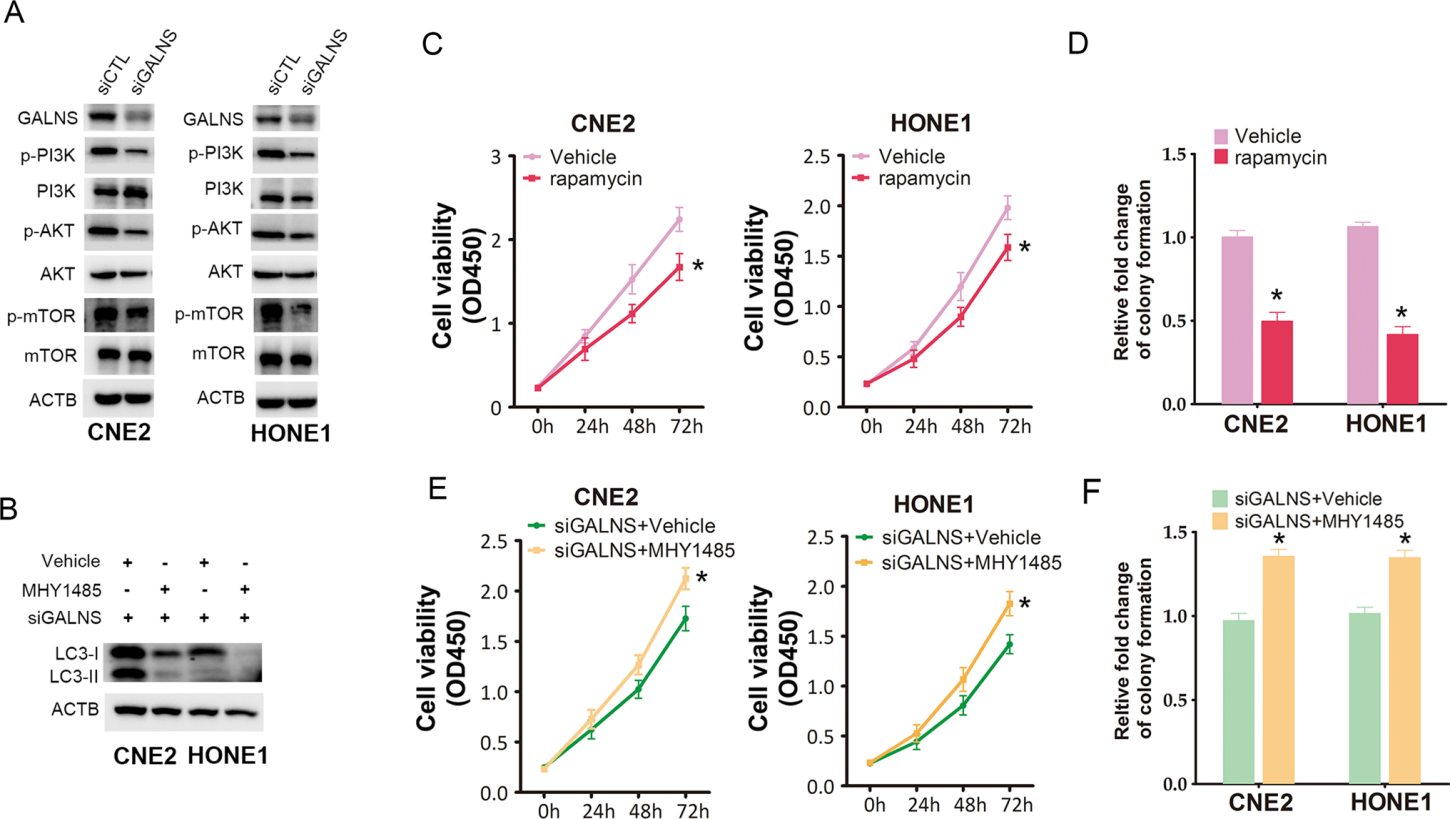
The PI3K-AKT-mTOR signaling is required for autophagy induced by GALNS knockdown. **A** Immunoblot showing expression of the PI3K–AKT–TOR pathway proteins in CNE2 and HONE1 cells transfected with siCTL or siGALNS. **B** Immunoblot showing LC3-II expression in GALNS-knockdown CNE2 or HONE1 cells treated with MHY1485. **C** Viability of rapamycin-treated CNE2 and HONE1 cells. **D** Number of colonies formed by rapamycin-treated CNE2 and HONE1 cells. **E** Viability of GALNS-knockdown CNE2 or HONE1 cells treated with MHY1485. **F** Number of colonies formed by GALNS-knockdown CNE2 or HONE1 cells treated with MHY1485. The data represent the mean ± SEM of at least three independent experiments. *p < 0.05, versus vehicle or siGALNS + vehicle

The PI3K–AKT–mTOR signaling pathway is the central regulator of autophagy. To determine whether PI3K–AKT–mTOR signaling also initiates autophagy in response to GALNS silencing, we analyzed the expression levels of the relevant proteins in control and GALNS-knockdown CNE2 and HONE1 cells. As shown in Fig. 5A, GALNS siRNA led to dephosphorylation of PI3K, AKT and mTOR. Furthermore, the mTOR activator MHY1485 attenuated autophagy induced by GALNS silencing in the NPC cells (Fig. 5B), and also enhanced the viability of the GALNS-knockdown cells (Fig. 5E). In addition, pretreatment with MHY1485 also increased the number of colonies formed by the NPC cells transfected with GALNS siRNA (Fig. 5F). On the other hand, inhibition of mTOR activity by rapamycin significantly decreased cell viability and colony formation in the CNE2 and HONE1 cells (Fig. 5C, D). Moreover, mTOR knockdown also suppressed NPC cell viability (Additional file 1: Figure S3A and B). Next, MK2206 (AKT inhibitor) or LY294002 (PI3K inhibitor) suppressed NPC cell viability (Additional file 1: Figure S4A and B). Taken together, these data suggested that GALNS knockdown suppressed NPC cell growth by inducing autophagy via inactivation of the PI3K–AKT–mTOR pathway.

## Discussion

NPC is a common malignancy that originates in the epithelium of the nasopharynx, and is associated with a low survival rate in southern China and Southeast Asia. Therefore, it is crucial to elucidate the mechanism underlying NPC development in order to identify novel therapeutic targets and improve the survival outcomes. In this study, we found that GALNS was highly expressed in NPC tissues, and its silencing in NPC cell lines inhibited their growth in vitro and in vivo via autophagy induction through the PI3K–AKT–mTOR signaling pathway. Therefore, our findings indicate that GALNS is a novel therapeutic target for NPC.

GALNS is a member of the lysosomal enzyme family, which hydrolyzes 6-sulfate groups from chondroitin sulfate and keratan sulfate. GALNS deficiency is associated with the autosomal-recessive mucopolysaccharide storage disorder [7, 25]. Studies increasingly show that GALNS might also contribute to cancer development. Wintergerst et al. found that GALNS is a functional gene and is associated with poor clinical outcomes of head and neck squamous cell carcinoma following chemo(radio)therapy [9]. Bhattacharyya et al. reported increased activity of GALNS in human prostate cancer tissues, which was associated with the increased Wnt/β-catenin signaling in human prostate stem cells [8]. More recently, Ho et al. demonstrated that GALNS is a potential diagnostic biomarker for multiple cancers [10]. However, the potential role and mechanism of GALNS in the development of NPC are still unknown. In this study, we found that GALNS mRNA and protein levels were significantly higher in NPC cell lines and tissues compared to the normal nasopharyngeal counterparts. Furthermore, silencing GALNS suppressed the growth of NPC cells in vitro and resulted in smaller tumors in vivo, indicating that GALNS functions as an oncogene in NPC.

Autophagy plays a complex role in tumor genesis and progression, and acts a double-edged sword depending on the type and stage of cancer [26–28]. Several studies have shown that autophagy can promote cancer cell survival in response to starvation, chemotherapy and hypoxia [29, 30]. Macintosh et al. reported that autophagy promoted glioma cell invasion in a 3D organotypic model [31]. In a previous study, we found that increased autophagy flux in adriamycin-resistant MCF-7/ADM cells contributed to the chemoresistance [32]. On the other hand, autophagy is also known to act a tumor suppressor [30]. For instance, Pang et al. showed that SCA promoted apoptosis of breast cancer cells by inducing autophagy [33]. We also showed previously that TRPV4 silencing induced colon cancer cell death through autophagy [34]. Furthermore, Yang et al. demonstrated that a Chinese herbal formula Qing Yan Li Ge Tang induced autophagic death in NPC cells [35]. Physcion, a naturally occurring anthraquinone derivative, can also trigger pro-apoptotic autophagy and lead to cell death [36]. In addition, overexpression of miR106A-5p promoted the malignant phenotype of NPC cells by inhibiting autophagy via the suppression of BTG3 [23]. Studies show that impaired autophagy is involved in chemo/radio-therapy resistance of NPC cells [37, 38]. However, the impact of GALNS-regulated autophagy on NPC progression and the underlying mechanisms remain to be elucidated. In this study, we found that GALNS knockdown increased LC3-II expression in NPC cells, indicating that it plays a key role in the induction of autophagy. Moreover, inhibition of autophagy neutralized the anti-proliferative effects of GALNS silencing in the NPC cells. These findings suggest that downregulation of GALNS suppresses the malignant phenotype of NPC cells via induction of autophagy.

The mTOR signaling pathway regulates autophagy by targeting the ULK1–FIP200 complex [39, 40]. PI3K/AKT pathway acts upstream of mTOR, and has a negative effect on autophagy [41, 42]. PI3K is an intracellular phosphatidylinositol kinase that phosphorylates phosphatidylinositol-(4,5)-bisphosphate to phosphatidylinositol-(3,4,5)-trisphosphate (PIP3) [43, 44], which then binds to the pleckstrin homology domain of AKT, resulting in its activation by phosphorylation [45]. The phosphorylated AKT then activates the downstream mTOR to initiate autophagy. Consistent with these findings, we found that GALNS knockdown dephosphorylated PI3K, AKT and mTOR in NPC cells. The exact mechanism through which GALNS activates PI3K remains to be elucidated. To determine whether GALNS silencing-induced autophagy is dependent on the PI3K/AKT/mTOR signaling pathway, we examined the effect of the pharmacological activation and inhibition of mTOR. While the mTOR activator MHY1484 inhibited autophagy and increased viability of the GALNS-knockdown NPC cells, inhibition of mTOR by rapamycin suppressed NPC cell growth. Thus, PI3K/AKT/mTOR signaling pathway is essential for GALNS silencing-induced autophagy in NPC cells. In agreement with our findings, Saiki et al. reported that caffeine induced cell death by upregulating autophagy via the PI3K/AKT/mTOR signaling pathway [45]. Rapamycin was clinically indicated for patients undergoing kidney transplantation to prevent organ rejection [46]. An accumulating evidences indicated that targeting PI3K/AKT/mTOR pathway by Rapamycin inhibits NPC cell development in vitro, suggesting that a potential application of Rapamycin for the clinical treatment of NPC [47].

In conclusion, our findings reveal a novel role of GALNS in regulating NPC progression. GALNS is overexpressed in NPC tissues, and promotes tumor growth via the PI3K/AKT/mTOR signaling pathway. Thus, GALNS is a potential therapeutic target for NPC and warrants further investigation.

### Supplementary Information

**Additional file 1: Figure S1.** The relative GALNS mRNA expression in the TCGA RNA-seq database. *** p < 0.001, versus Normal. **Figure S2.** A, Viability of C666-1 cells transfected with siCTL or siGALNS. B, Relative fold change of colony formation in C666-1 cells transfected with siCTL or siGALNS. The data represent the mean ± SEM of at least three independent experiments. * p < 0.05, versus siCTL. **Figure S3.** A and B, Viability of CNE2 and HONE1 cells transfected with siCTL or simTOR. The data represent the mean ± SEM of at least three independent experiments. * p < 0.05, versus siCTL. **Figure S4.** A, Viability of CNE2 and HONE1 cells pretreated with Vehicle or MK2206. B, Viability of CNE2 and HONE1 cells pretreated with Vehicle or LY294002. The data represent the mean ± SEM of at least three independent experiments. * p < 0.05, versus Vehicle.

## Data Availability

The datasets generated during and/or analysed during the current study are available from the corresponding author on reasonable request.
