# Supplementary material for: Identification of galactosamine-(N-acetyl)-6-sulfatase (GALNS) as a novel therapeutic target in progression of nasopharyngeal carcinoma
Source: Discov Oncol. 2023 Sep 14;14:171. doi: 10.1007/s12672-023-00782-4 (PMC10501037; doi:10.1007/s12672-023-00782-4)
Supplement: Supplementary file 1 — Additional file 1: Figure S1. The relative GALNS mRNA expression in the TCGA RNA-seq database. *** p < 0.001, versus Normal. Figure S2. A, Viability of C666-1 cells transfected with siCTL or siGALNS. B, Relative fold change of colony formation in C666-1 cells transfected with siCTL or siGALNS. The data represent the mean ± SEM of at least three independent experiments. * p < 0.05, versus siCTL. Figure S3. A and B, Viability of CNE2 and HONE1 cells transfected with siCTL or simTOR. The data represent the mean ± SEM of at least three independent experiments. * p < 0.05, versus siCTL. Figure S4. A, Viability of CNE2 and HONE1 cells pretreated with Vehicle or MK2206. B, Viability of CNE2 and HONE1 cells pretreated with Vehicle or LY294002. The data represent the mean ± SEM of at least three independent experiments. * p < 0.05, versus Vehicle. [file 12672_2023_782_MOESM1_ESM.docx]

**Additional Material**


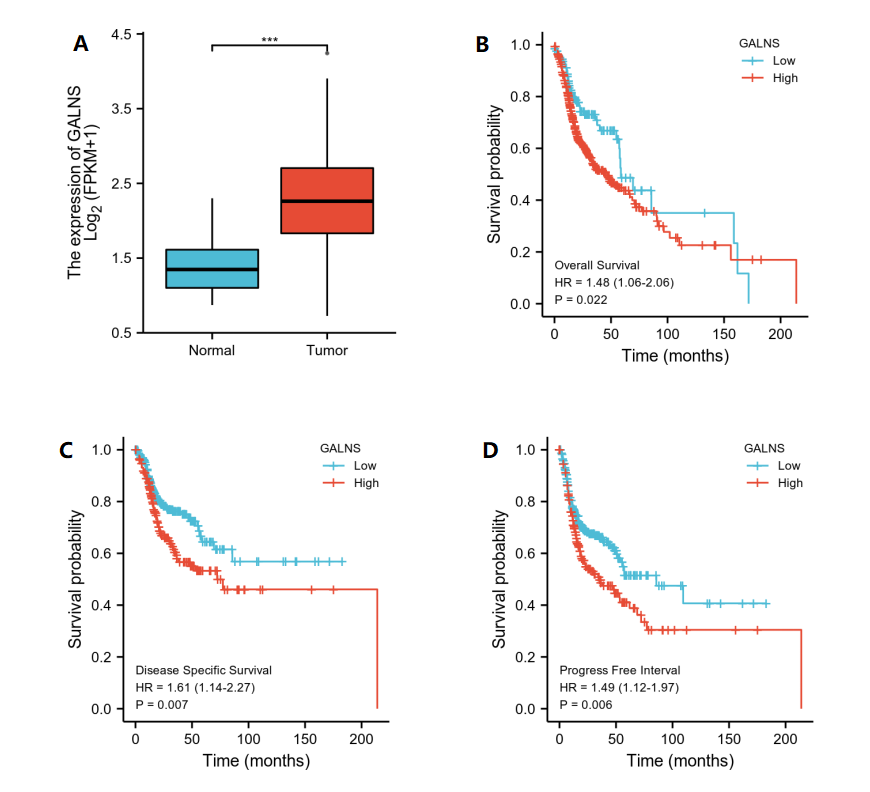


**Figure S1.** The relative GALNS mRNA expression in the TCGA RNA-seq database. *** p<0.001, versus Normal.


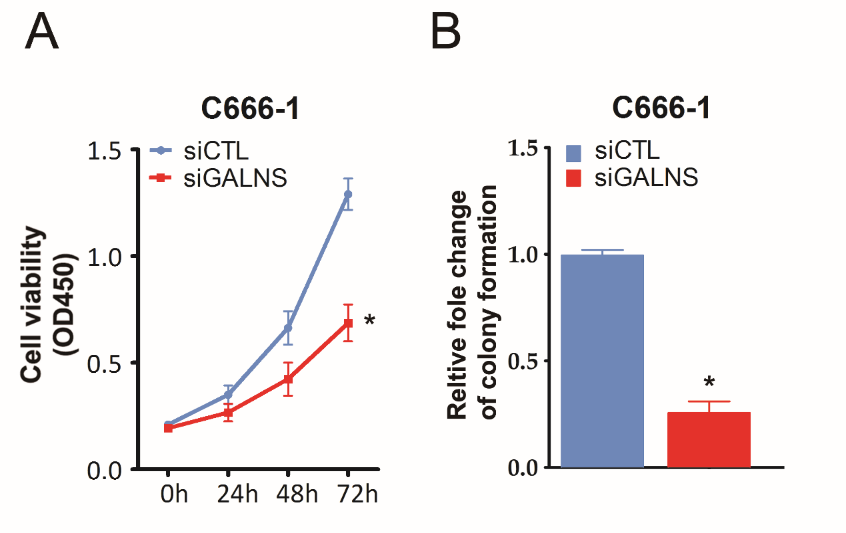


**Figure S2.** A, Viability of C666-1 cells transfected with siCTL or siGALNS. B, Relative fold change of colony formation in C666-1 cells transfected with siCTL or siGALNS. The data represent the mean ± SEM of at least three independent experiments. * p<0.05, versus siCTL.


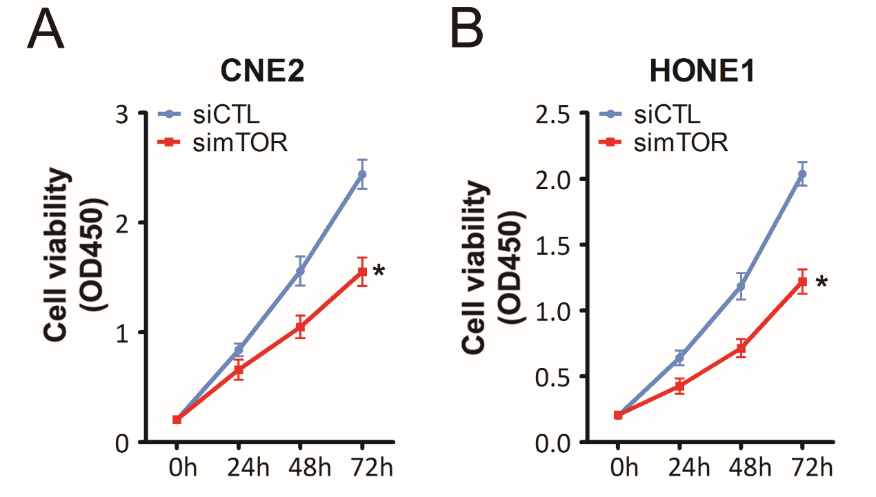


**Figure S3.** A and B, Viability of CNE2 and HONE1 cells transfected with siCTL or simTOR. The data represent the mean ± SEM of at least three independent experiments. * p<0.05, versus siCTL.


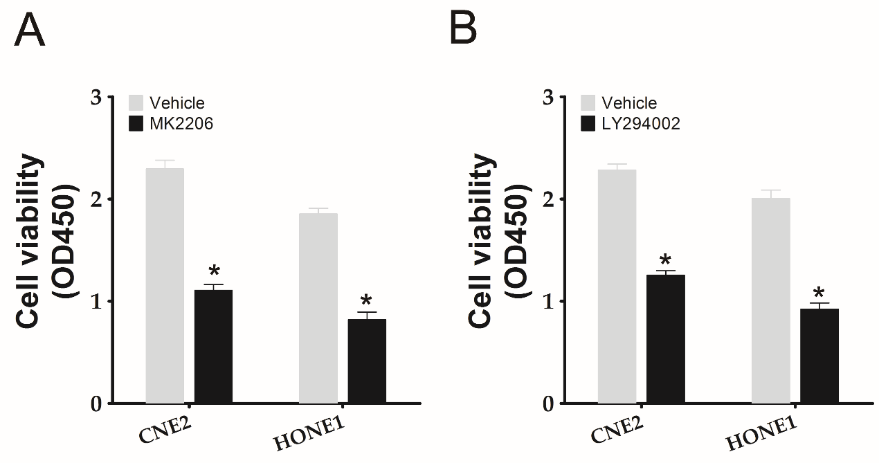


**Figure S4.** A, Viability of CNE2 and HONE1 cells pretreated with Vehicle or MK2206. B, Viability of CNE2 and HONE1 cells pretreated with Vehicle or LY294002. The data represent the mean ± SEM of at least three independent experiments. * p<0.05, versus Vehicle.
